# Educational Intervention Improves Anticoagulation Control in Atrial Fibrillation Patients: The TREAT Randomised Trial

**DOI:** 10.1371/journal.pone.0074037

**Published:** 2013-09-09

**Authors:** Danielle E. Clarkesmith, Helen M. Pattison, Gregory Y. H. Lip, Deirdre A. Lane

**Affiliations:** 1 University of Birmingham Centre for Cardiovascular Sciences, City Hospital, Birmingham, United Kingdom; 2 School of Health and Life Sciences, Aston University, Birmingham, United Kingdom; Maastricht University Medical Center, Netherlands

## Abstract

**Background:**

Stroke prevention in atrial fibrillation (AF), most commonly with warfarin, requires maintenance of a narrow therapeutic target (INR 2.0 to 3.0) and is often poorly controlled in practice. Poor patient-understanding surrounding AF and its treatment may contribute to the patient’s willingness to adhere to recommendations.

**Method:**

A theory-driven intervention, developed using patient interviews and focus groups, consisting of a one-off group session (1–6 patients) utilising an “expert-patient” focussed DVD, educational booklet, self-monitoring diary and worksheet, was compared in a randomised controlled trial (ISRCTN93952605) against usual care, with patient postal follow-ups at 1, 2, 6, and 12-months. Ninety-seven warfarin-naïve AF patients were randomised to intervention (n=46, mean age (SD) 72.0 (8.2), 67.4% men), or usual care (n=51, mean age (SD) 73.7 (8.1), 62.7% men), stratified by age, sex, and recruitment centre. Primary endpoint was time within therapeutic range (TTR); secondary endpoints included knowledge, quality of life, anxiety/depression, beliefs about medication, and illness perceptions.

**Main Findings:**

Intervention patients had significantly higher TTR than usual care at 6-months (76.2% vs. 71.3%; p=0.035); at 12-months these differences were not significant (76.0% vs. 70.0%; p=0.44). Knowledge increased significantly across time (F (3, 47) = 6.4; p<0.01), but there were no differences between groups (F (1, 47) = 3.3; p = 0.07). At 6-months, knowledge scores predicted TTR (r=0.245; p=0.04). Patients’ scores on subscales representing their perception of the general harm and overuse of medication, as well as the perceived necessity of their AF specific medications predicted TTR at 6- and 12-months.

**Conclusions:**

A theory-driven educational intervention significantly improves TTR in AF patients initiating warfarin during the first 6-months. Adverse clinical outcomes may potentially be reduced by improving patients’ understanding of the necessity of warfarin and reducing their perception of treatment harm. Improving education provision for AF patients is essential to ensure efficacious and safe treatment.

The trial is registered with Current Controlled Trials, ISRCTN93952605, and details are available at www.controlled-trials.com/ISRCTN93952605.

## Introduction

Oral anticoagulation (OAC) significantly reduces the risk of stroke in atrial fibrillation (AF) patients [1]. Until recently the mainstay of OAC therapy was vitamin K antagonists, most commonly with warfarin. However, warfarin requires AF patients to maintain a narrow therapeutic range (INR range 2.0 to 3.0). Analyses of a cohort of AF patients from the General Practice Research Database found that overall patients spent 63% of their time in therapeutic range (TTR) [2]. Patients that spent at least 70% of their time in therapeutic range had a 79% reduced risk of stroke compared to patients with ≤30% of time in range [2].

Research suggests where patients have a greater knowledge of warfarin therapy, INR values are more often within the target therapeutic range [3]. However, AF patients often exhibit limited knowledge of their condition and their anticoagulant therapy [3–6]. A previous brief educational intervention demonstrated a significant improvement in the awareness of target therapeutic INR (p<0.0001) and factors which may affect INR levels (p=0.005) when assessed six weeks later [4]. An individual patient-data meta-analysis of self-management trials demonstrated significant improvements in TTR and a significant reduction in thromboembolic events with self-monitoring but no difference in the risk of major bleeding or death [7]. Whilst self-management improves anticoagulation control, this may not be a feasible option for the majority of the patients requiring anticoagulation, due to the training required [8]. In addition, the associated costs of self-monitoring may prevent wide-scale uptake [9], particularly with the arrival of novel oral anticoagulants that do not require monitoring [9,10].

One study suggests that education alone may also have a significant impact on time in therapeutic range (TTR) [11], but the mechanisms by which education improves patient adherence to the warfarin regimen are unclear. Evidence does suggest that patients’ lack of knowledge surrounding their condition and treatment presents a key barrier to uptake and adherence [3,4]. Further, patients’ perceptions of their illness suggest that AF patients may formulate inaccurate perceptions [12], which may lead to intentional non-adherence to medication.

Patient barriers to optimal OAC treatment are complex. There are several reasons why patients may choose not to take OAC or why they may not adhere to medication and lifestyle recommendations. Factors include the presentation of risk information and how this is communicated; particularly the ‘framing’ of the message as a positive or negative choice [13]. The evidence suggests the majority of AF patients are unaware that they are at risk of stroke [14]. Patient’s lack of knowledge surrounding their treatment and condition, coupled with the potential burden of a complex regime may be barriers to treatment uptake and adherence.

In this randomised controlled trial, we examined the impact of a disease-specific theory-driven educational intervention on TTR and other outcomes including patient knowledge, illness perceptions, beliefs about medications, and perceived benefits and burdens of warfarin, compared to patients with AF receiving usual care alone.

## Methods

### Study design

All patients attending a specialist AF clinic or local anticoagulation outpatient clinic, with documented AF, who were warfarin-naïve (having never taken warfarin) and accepting of OAC therapy were approached to participate in the TREAT (**TR**ial of an **E**ducational intervention on patients’ knowledge of Atrial fibrillation and anticoagulant therapy, INR control, and outcome of **T**reatment with warfarin) study. The trial is registered with Current Controlled Trials, ISRCTN93952605, and details are available at www.controlled-trials.com/ISRCTN93952605. The protocol for this trial has been previously published [15]. The protocol and supporting CONSORT checklist are available as supporting information; see Checklist S1 and Protocol S1. The TREAT intervention was designed to provide intensive education for AF patients newly prescribed warfarin and is based on psychological theory and key clinical guidelines [16,17]. The purpose of the study was to evaluate the use of a one-off theory driven educational intervention on the primary endpoint of the proportion of time spent within therapeutic INR range (TTR). Secondary outcomes included patient knowledge, illness perceptions, anxiety and depression, beliefs about medication, and health-related quality of life.

Patients were randomised to receive either the intervention or usual care. Patients were excluded from participation if they were aged <18 years old, had any contraindication to warfarin, had previously received warfarin, had valvular heart disease, were cognitively impaired or had dementia, were unable to speak or read English, or had any disease likely to cause their death within the subsequent 12 months.

### Materials and procedure

Patients received an information sheet detailing the study and provided written informed consent. The research protocol and amendments were approved by the Black Country Local Ethics Research Committee. A telephone or face-to-face interview permitted the collection of socio-demographic data including: age, gender, occupational status, number of years in education, postcode (for socio-economic status index) and ethnicity. Further interrogation of hospital records allowed for collection of baseline clinical measures (e.g. body mass index (BMI), AF history, ECG, blood pressure, left ventricular function, medication) and verification of socio-demographic information. Patients completed a series of postal questionnaires on five occasions: baseline, 1, 2, 6 and 12 months. The Beliefs about Medication Scale is an 18-item questionnaire assessing patient’s specific beliefs about their prescribed medication, including concerns and perceived necessity of treatment, as well as their beliefs about medication harm and overuse in general. Scores for each subscale are summed and divided by the number of items, giving a score of 5 to 25 [18]. The Hospital Anxiety and Depression Scale, has two separate subscales assessing anxiety and depression, scores on each scale are summed with a score of ≥8 on either sub-scale indicating a case [19]. The Brief Illness Perception Questionnaire, based on the Common Sense Model, assesses patients perceptions surrounding their illness (AF) including (i) *identity*- symptoms patients associate with the illness and what they attribute to the illness; (ii) *consequences*- expected physical, social and economic implications; (iii) *timeline* - acute, chronic or cyclical duration; (iv) *causes*- personal ideas about causes; and (v) *cure/control*- the extent to which a patient believes they will recover from or control their illness [20]. The Atrial Fibrillation Quality of Life (QoL) Questionnaire, an 18-item health-related QoL scale, was used to assess psychological, physical, sexual and global quality of life [21]. Values close to zero show a worse health state, while values close to 100 indicate a better health state. The Patient Knowledge Questionnaire (14 items) was previously designed and piloted by our research group to assess patients’ knowledge of their condition, AF, and anticoagulant treatment [4]. Nine items are scored to give a total knowledge score; the remaining five items are qualitative and are coded to give further qualitative insight into patients’ knowledge of AF and OAC.

### Endpoints

The primary endpoint is the proportion of time spent in therapeutic INR range, INR2.0 to 3.0, at 6 and 12 months. Every INR result, from baseline to the end of the study (12-months), was recorded on an INR log sheet. INR readings were undertaken by the anticoagulation service at each hospital (independent of the study) to ensure the findings were as ‘naturalistic’ as possible. The proportion of time each patient spent in the therapeutic INR range (2.0 to 3.0) was calculated by the method of linear interpolation (Rosendaal method) [22] using data from month one to 12 (to allow attainment of the correct dose of warfarin during the first four weeks) to give the time spent within target therapeutic range (TTR). The following secondary endpoints were also examined, patient’ knowledge, beliefs about medication, anxiety and depression, illness representations, and health-related QoL. Hospital admissions and clinical outcomes (death, thromboembolism, stroke, major bleeding, and myocardial infarction) during the first 12 months were obtained from patients’ hospital records.

### Randomisation and masking

A computer generated list stratified by (a) age (<70 and ≥70 years)/sex and (b) specialist AF clinic versus ‘general’ cardiology clinic, in blocks of four, randomised patients on an individual basis to receive either ‘usual care’ or the intensive educational intervention, in addition to ‘usual care’. The randomisation schedule was designed by an independent trials unit and the random allocation was obtained by the researcher telephoning an associate researcher (not involved in the data collection or data entry). A third researcher (not involved in the data analysis or intervention delivery) matched patient identification numbers with randomisation codes and checked the completeness of follow-up questionnaires, and contacted patients via telephone if any questions were not completed. Patients who were unable to attend the intervention education session within the specified time period (up to one month after warfarin initiation) crossed over to the usual care arm (n=3). The randomisation codes and data will be made available upon request.

### Intervention development and delivery

The intervention was designed utilising the ‘Necessity-Concerns Framework’ to understand the key beliefs which influence whether patients adhere to prescribed treatment or not [23]. The model suggests that patients hold beliefs about the necessity (specific-necessity) and concerns (specific-concerns) surrounding prescribed medication. The model also describes general beliefs about medication, assessing beliefs that medicines are addictive and harmful (general-harm), and that medicines are over-prescribed by doctors (general-overuse). The Common Sense Model (CSM) also provided guidance [20]. Utilising this model the intervention integrated each of the five components that contribute to the formation of a patient’s perception of their illness (AF). For example, to ensure patients ‘understand the consequences’ the intervention focussed on the physical, social and economic implications of AF during the intervention.

### TREAT intervention

Patients attended one group session [between 1–6 patients] for one hour where they were shown a DVD of information about the need for OAC, the risks and benefits associated with OAC therapy, potential interactions with food, drugs, and alcohol, and the importance of monitoring, and control of their INR. The intervention was developed following discussions with AF patient focus groups and patient interviews, and was communicated in a variety of ways [i.e. by expert patients, a cardiology consultant, other healthcare professional, and examples of food/alcohol dietary components with educational information as a voiceover script]. Patients were encouraged to ask questions and complete a worksheet-based exercise following each 10 minute DVD section.

### Usual care

All patients received the standard ‘yellow booklet’ to identify that they are taking OAC therapy. This book contains generic information for all patients taking OAC (including deep vein thrombosis, pulmonary embolism etc) and includes key safety information including dietary advice (a brief paragraph instructing patients not to miss meals and keep their diet stable), medication (to inform GP/physician if they start a new medication) and emergency contact information.

Three patients randomised to receive the intervention could not attend the intervention within one month of warfarin initiation. Two patients were ill during the month following warfarin commencement and one patient could not be contacted. Those patients that did not receive the intervention were included in the usual care arm for the on-treatment analyses.

### Sample size calculation

Power for the primary endpoint was calculated based on data from a secondary analysis of TTR from the ACTIVE-W cohort by Connolly et al [24]. The power calculation assumes that usual care patients would have a mean TTR of 58% with a standard deviation (SD) of 7.5. We hypothesised a 6% improvement in mean TTR in the intervention group with a similar SD. In order for this improvement in TTR to be statistically significant with a 1-beta power of 0.99 and alpha=0.01, a sample size of 156 subjects in two equal groups of 78 is needed, to allow for a 20% attrition rate.

For the secondary endpoint of improvement in knowledge following the intervention, the sample size was calculated based on a study by Khan et al [11]. A sample size of 100 patients (50 in each group), allowing for a 20% attrition rate in the completion of the questionnaires, will have at least 80% power to detect an 18.5% increase in knowledge about the condition and factors affecting INR control between baseline and follow-up.

### Statistical analysis

Data were analysed using IBM SPSS Statistics (Version 21.0). All tests were two tailed, where p-values ≤0.05 they were considered statistically significant. Descriptive statistics are presented for baseline demographic and clinical information. Categorical variables were analysed using the chi-square statistic and the Fisher exact test was used where there were expected frequencies of less than five in any cell. Continuous variables were compared using independent t-tests. Where data were not normally distributed a Mann Whitney-U test was used. All data were analysed by intention-to-treat. On-treatment analyses are also provided for TTR. Three patients who were randomised to the intervention group but could not receive the intervention within one month of initiating warfarin were included in the usual care group in the on-treatment analysis. The primary endpoint, TTR, was determined by the method of linear interpolation using the Rosendaal method [22], using INR data from months one to 12. Differences in TTR between the two groups were examined using the Mann Whitney-U test and are reported as median and inter-quartile range. Data for the secondary endpoints of patient’ knowledge, beliefs about medication, anxiety and depression, illness representations, and health-related QoL at the five time-points (baseline, 1, 2, 6, and 12 months) are presented graphically to illustrate the change in these variables over time between the intervention and usual care groups. Assessment of the impact of the intervention compared to usual care at 6 and 12 months was undertaken using the change from baseline for each of the secondary endpoints, with separate analyses for the 6 and 12 month time-points, using the Mann Whitney-U test and employing a more conservative p-value (≤0.01) to adjust for a Type I error due to multiple statistical comparisons. To measure the change in variables across time (at five time points including baseline, one, two, six and 12 month follow-ups) and between groups (intervention and usual care groups) for those who completed the questionnaires on all occasions (n=29), data for each psychological outcome was entered into separate two-factor mixed ANOVA analyses, where ‘group’ is assumed fixed and ‘time-point’ is assumed random. Where the assumptions of the test were violated (Mauchley’s test of sphericity p<0.05), a more conservative p-value was reported (Greenhouse-Geisser). Multiple regression analyses were used to determine predictors of TTR. 

## Results

**Figure 1:**
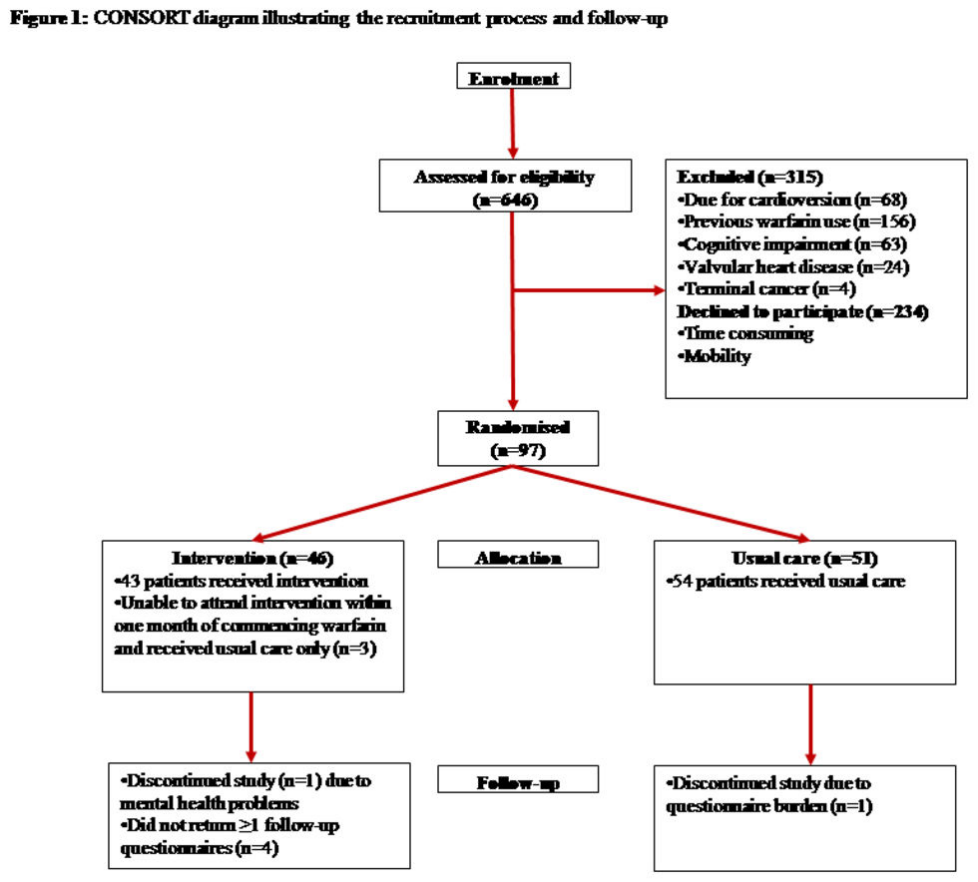
CONSORT diagram illustrating recruitment process and follow-up.

Ninety-seven patients participated in the study. Forty-six patients were randomised to the intervention group and 51 to usual care (see Figure 1). There were no significant differences between the intervention and usual care groups on any baseline demographic or clinical characteristics (see Table 1). The mean (SD) age of the total cohort was 72.9 (8.2) years. Almost half (48.4%) of the patients were aged 65-74 years old, 64.9% were male, and almost all (99.0%) were White British, Irish or European. The median (IQR) years in education were 10 (9.5 to 12.0). The median (IQR) CHADS_2_ score for the total cohort was 2 (1–3). There were no significant differences in baseline prescribed medication. Three patients were unable to attend the intervention within one month of commencing warfarin and they received usual care only. There were no significant differences in demographic or clinical variables between the treatment groups in the on-treatment analyses (data not shown).

**Table 1:** Baseline demographics of the whole cohort and by randomisation arm.

Demographic characteristics Mean (SD), n (%)	All participants n=97	Intervention n=46	Usual Care n=51	p-value
Age, years	72.9 (8.2)	72.0 (8.2)	73.7 (8.1)	0.32
Age, years				
<65	14 (14.4)	6 (13.0)	8 (15.7)	
65-74	47 (48.5)	26 (56.5)	21 (41.2)	0.31
≥75	36 (37.1)	14 (30.4)	22 (43.1)	
Sex				
Males	63 (64.9)	31 (67.4)	32 (62.7)	0.79
Females	34 (35.1)	15 (32.6)	19 (37.3)	
Ethnicity				
White ‡	96 (99.0)	46 (100.0)	50 (98.0)	1.00
Years in education †	10	10	10	0.92
	(9.5-12.0)	(9.75-12.0)	(9.25-12.0)	
Socio-economic status†	20.9	22.3	20.4	0.55
	(9.3-37.1)	(9.5-38.1)	(8.9-34.7)	
Body Mass Index (kg/m^2^)	28.4 (5.4)	28.9 (5.6)	28.0 (5.2)	0.39
Type of AF				
Paroxysmal	30 (30.9)	13 (28.3)	17 (33.3)	
Persistent	22 (22.6)	11 (23.9)	11 (21.6)	0.37
Permanent	24 (24.7)	15 (32.6)	9 (17.6)	
Duration of known AF in months †	3.0 (1.0-14.0)	3.0 (1.0-19.5)	2.0 (1.0-12.0)	0.87
Alcohol units per week †	4.0 (0-12)	4.0 (0-14)	4.0 (0-10)	0.43
Smoking status				
Current smoker	4 (4)	3 (6.5)	1 (2.0)	
Ex-smoker	46 (46.5)	19 (41.3)	22 (43.1)	0.31
Non smoker	46 (46.5)	24 (52.2)	27 (52.9)	
*CHADS*_*2*_ * risk factors*				
**C**ongestive Heart Disease/ LV dysfunction	17 (17.5)	9 (19.6)	8 (15.7)	0.90
Hypertension	65 (67.0)	33 (71.7)	32 (62.7)	1.00
**A**ge ≥75	36 (37.1)	14 (30.4)	22 (43.1)	0.31
Diabetes mellitus	15 (15.5)	7 (15.2)	8 (15.7)	0.95
**S**troke	6 (6.2)	1 (2.2)	5 (9.8)	0.19
Transient ischaemic attack	10 (10.3)	5 (10.9)	5 (9.8)	1.00
Total CHADS_2_ score†	2 (1-3)	2 (1-2)	2 (1-3)	0.28
*Baseline medication*				
Calcium channel blockers	23 (23.7)	9 (19.6)	14 (27.5)	0.47
Beta-blocker	36 (37.1)	20 (43.5)	16 (31.4)	0.34
Anti-platelet	11 (11.3)	6 (13.0)	5 (9.8)	0.88
ACE- inhibitor	40 (41.2)	23 (50.0)	17 (33.3)	0.16
Statin	34 (35.1)	17 (37.0)	17 (33.3)	0.92
Digoxin	16 (16.5)	7 (15.2)	9 (17.6)	0.93
Diuretic	31 (32.0)	15 (32.6)	16 (31.4)	1.00

† Median (IQR); ‡ Only one patient in the study was not White (British/Irish/European ACE, angiotensin converting enzyme; CHADS_2_, Congestive heart failure/left ventricular dysfunction, Hypertension, Age ≥75 years, Diabetes mellitus (1 point for presence of each), Stroke/Transient ischaemic attack (2 points); LV, left ventricular;

### Time within therapeutic range

In the intention-to-treat analysis, the intervention group spent significantly more time in therapeutic INR range during the first 6 months of OAC initiation than patients receiving usual care (76.2% vs. 71.3% respectively; p=0·035) (see Table 2). Differences between the groups remained at the 12 month follow-up (76.0% vs. 70.0%; p=0.44), but were non-significant (see Table 2). At the 6 and 12 month follow-ups patients in the usual care group spent more time with a sub-therapeutic INR (INR<2·0) than patients in the intervention group but these differences were not significant. Both the intervention and usual care groups attended the anticoagulant clinic a similar number of times at both the 6 and 12 month follow-ups. The results were very similar for the on-treatment analyses however, patients receiving usual care spent significantly more time with sub-therapeutic INRs in the first 6 months than those receiving the intervention (see Table 2).

**Table 2:** The proportion of time spent within therapeutic range at 6 and 12 months stratified by treatment group.

		**6 months**				**12 months**		
**Intention-to-treat analyses**								
Median (IQR)	**Intervention**		**Usual care**		p-value	**Intervention**	**Usual care**	p-value
	**(n=42**)		**(n=49**)			**(n=37**)	**(n=41**)	
Overall TTR	76.2		71.3		**0.035**	76.0	70.0	0.44
	(64.1-97.3)		(51.2-84.7)			(60.5-85.0)	(62.0-79.0)	
Proportion of INR>3.0	0 (0-20.2)		8.9 (0-22.6)		0.17	12.0 (0.5-19.0)	10.0 (0-23.0)	0.91
Proportion of INR<2.0	9.7 (0-28.9)		19.5 (2.6-33.2)		0.08	12.0 (5.0-22.5)	14.0 (6.0-24.0)	0.52
Mean (SD) number of INR visits	6.9 (2.3)		7.0 (2.9)		0.84	12.9 (4.0)	12.7 (3.8)	0.79
**On-treatment analyses**								
Median (IQR)		**Intervention**	**Usual care**	p-value		**Intervention**	**Usual care**	p-value
		**(n=39**)	**(n=52**)			**(n=35**)	**(n=43**)	
Overall TTR		82.5	68.9	**0.009**		76.0	69.0	0.21
		(66.7-97.6)	(51.1-83.0)			(61.0-86.0)	(60.0-78.0)	
Proportion of INR>3.0		0 (0-20.0)	7.0 (0-22.0)	0.22		10.0 (0-20.0)	13.0 (0.22.0)	0.79
Proportion of INR<2.0		8.45 (0-27.4)	19.8 (3.4-33.3)	**0.021**		12.0 (4.0-22.0)	15.0 (7.0-24.0)	0.26
Mean (SD) number of INR visits		6.7 (2.0)	7.2 (3.0)	0.35		12.5 (3.5)	13.0 (4.2)	0.57

INR = international normalised ratio, IQR = inter-quartile range; SD = standard deviation; TTR = time in therapeutic range

INR data were not available on six (6.2%) patients at the 6 month follow-up and on 19 (19.6%) patients at the 12 month follow-up

Key baseline demographics, sex, age and years spent in education were included in a multiple linear regression model to predict TTR at 6 months. TTR was higher in women than in men (74.5% (95% CI 57.7-97.3) vs. 72.3% (95% CI 59.3-86.8), respectively; z=-0.913, p=0.36), but differences were not significant. There were no significant differences between age categories (<65, 65-74, ≥75 years; p=0.48), or years spent in education (p=0.31). Years spent in education was the only factor that predicted 12 month TTR (p=0.01).

### Patient’s knowledge

Both groups of patients demonstrated good levels of knowledge regarding AF at baseline, as they answered six out of nine questions correctly on average. Knowledge scores increased slightly in the intervention group over time, remaining at a median score of seven at all subsequent time-points. There were significant improvements in knowledge across time (F(1,22) = 4.5; p<0.04), but not between groups.

A Spearman’s correlation coefficient revealed that patient knowledge at baseline, one and two month follow-ups did not predict TTR, whereas knowledge at the six month follow-up weakly predicted overall TTR (r=0.245; p=0.04). There were no significant correlations between the 12-month TTR and knowledge scores at any time point.

### Change in psychological measures from baseline to 6 and 12 months

The change from baseline to 6 months and baseline to 12 months was compared between groups for all psychological variables (see Table S1). There was no significant change in any of the psychological variables between groups at either 6 or 12 months. Therefore, exploratory analyses were conducted to examine differences in the psychological variables over time between the groups in patients who completed the questionnaires at each time point to elucidate possible reasons for the differences in TTR between groups at 6 and 12 months. The number of patients completing questionnaires at 1, 2, 6 and 12 months was 72 (74.2%), 57 (58.8%), 61 (62.9%), and 53 (54.6%), respectively (see Table S2). However, only 29 (29.9%) patients completed ≥1 questionnaires at all time points (some did not complete all questionnaires). The results are reported below.

### Illness Perceptions

There were significant differences in the patients’ perception of the timeline of AF (whether patients perceive their AF duration is acute, chronic or cyclical in duration) across time (F(4,25) = 5.9; p<0.01), but no differences between groups. Patients’ perceived treatment control (F(4,25) = 2.7; p=0.05), emotional representation (F(4,26) = 3.1; p=0.04), and their illness coherence scores changed significantly over time (F(4,26) = 4.6; p<0.01), but there was no significant differences between groups. IPQ factors did not predict TTR at 6 or 12 months.

Patients in the intervention group scored higher on illness coherence, lower on emotional representation (how much their illness affected them emotionally), and lower on illness concern than the usual care group. However, none of the differences between groups reached statistical significance (see Figure S2 and Table S3).

### Quality of life

Patients in the intervention group scored lower at baseline on all QoL subscales, suggesting worse QoL than in the usual care group. QoL increased in the intervention group at the one month follow-up. At all subsequent follow-ups there were no significant differences in QoL scores between groups. There were no significant differences in QoL between or within groups (from baseline to 12 month follow-up) (see Figure S2).

### Beliefs about medication

There were significant differences between groups in the perception of the general harm of medication (F(1,28) = 4.4; p<0.05); the intervention group viewed medication as less harmful than the usual care group. There was also a significant interaction between group and time (F(4,28) = 2.7; p=0.03), but no significant changes across time in patients’ perception of general harm (see Figure S3).

There was also a significant interaction between group and time for patients’ perception of the general overuse of medication (F(4,28) = 2.4; p=0.04). The usual care group perceived medication as more overused by health care professionals than the intervention group. No significant differences between groups were evident in general overuse (p=0.06), or changes in scores across time. There were no significant differences between groups or across time in patient’s scores for the specific necessity of medication subscale. There was an interaction between time and group for patients’ concerns regarding medication (F(4,27) = 2.9; p=0.02); patients in the intervention group had fewer specific concerns about medication at all time-points other than at the 12 month follow-up. There were no significant differences between groups or across time for scores on the specific concerns subscale.

A multiple linear regression model found that perceived general harm of medication at 1 month was the only predictor of TTR at 6 months (F(1,72) = 4.08; p=0.048). A negative correlation (r=-0.241; p= 0.021) exists between general harm scores and TTR; suggesting that as the perceived general harm scores increased, TTR decreased. Baseline specific necessity subscale scores predicted 12 month TTR (F(1,96) = 3.88; p=0.05). One month scores for specific necessity (p=0.03), general harm (p=0.01) and general overuse (p=0.02) also predicted 12 month TTR (F(3,72) = 3.4; p=0.02). General harm (p=0.02) and general overuse (p=0.05) scores at the 6 month follow-up predicted 12 month TTR (F(2,61) = 3.2; p=0.05).

### Anxiety and Depression

At baseline, median anxiety scores for the total cohort were just below the cut-off for clinical significance (score of ≥8; see Table S3). At all subsequent follow-ups anxiety scores in both groups increased significantly. A similar pattern was exhibited with regard to depression. At baseline patients had relatively low depression scores [median (IQR) 4.9 (2–8)], but these scores increased significantly at each follow-up suggesting that over half of the patients in both groups were depressed. The prevalence of depression cases doubled from baseline to one month (25.5% to 55.6%), as did the prevalence of anxiety (41.5% to 95.4%).

There was a significant increase in anxiety scores across time (F(4,23) = 5.6; p<0.01), and significant differences between the intervention and usual care groups (F(1,23) = 4.7; p <0.05), but there was no significant interaction between time and group. There was also a significant increase in depression scores across time (F(4,23) = 14.4; p<0.01), but no significant differences in depression between groups or interaction between time and group (see Figure S4).

### Adverse events

Only eight adverse events occurred during the 12 month follow-up; seven in the usual care group (three ischaemic non-fatal strokes, two minor bleeding episodes, one major bleeding episode and one non-cardiac related death) and one event in the intervention group (peripheral embolism).

## Discussion

In this randomised trial, the TREAT intervention significantly improved patients’ warfarin control compared to usual care, evidenced by significantly more time spent in the therapeutic INR range at the 6 month follow-up. Patients in the intervention group had better TTR at 12 months than those receiving usual care only but these differences were not significant. This suggests greater adherence to medication and lifestyle recommendations in those patients receiving the TREAT intervention.

Two recent systematic reviews and meta-analyses [25,26], one examining supplemental education for patients taking OAC (for any indication) [25], and the other investigating educational and behavioural interventions on OAC therapy exclusively in AF patients [26], found that such interventions did not significantly improve TTR. However, both concluded that this was likely dependent on the considerable clinical and methodological heterogeneity of included studies and poorly designed interventions (which did not employ intensive education or behaviour change interventions designed to affect psychological outcomes and improve treatment adherence) rather than the lack of benefit of educational interventions *per se* on outcomes, and called for larger randomised trials with longer follow-up, in patients initiating OAC therapy, with clearly defined educational interventions [25,26].

To our knowledge only one randomised controlled trial has explored the impact of education alone on adherence in AF patients, and this trial did not exclusively target patients who were newly diagnosed with AF or who were warfarin naïve [11]. Further, Khan and colleagues did not account for psychological barriers to adherence, such as inaccurate perceptions of illness (including a poor understanding of the cause, consequences and timeframe of AF) or beliefs about medication. By increasing the provision of information to patients to help them to formulate accurate beliefs and perceptions surrounding AF and warfarin, patients maybe more able and willing to adhere, in the long-term, to treatment recommendations through better understanding of the condition and its’ treatment. The clinical implications of these findings are important as the effectiveness of oral anticoagulation treatment with warfarin is often undermined by low levels of adherence [27–30]. Maintaining the therapeutic INR range of 2·0 to 3·0 is imperative for stroke risk reduction and to reduce the risk of treatment-associated bleeding complications [2,27,31–33]. Evidence suggests that warfarin treatment offers no or limited clinical benefit (reduced stroke and mortality) unless a patient can maintain their therapeutic range for more than 71% of the time [2]; a target achieved by those in the intervention group at both 6 and 12 months. Thus, the use of a one-off theory-driven intervention could help to ensure that patients starting warfarin would establish and maintain ‘good’ INR control and therefore achieve the desired treatment benefit. Improvements to usual care TTR over time are likely caused by their experiences of the warfarin regime and even minor bleeding episodes.

Improving TTR in the first six months of treatment is essential, as it is this period of time where patients have the most unstable INR control and are most likely to discontinue therapy [2]. The non-significant difference in TTR between the intervention and usual care groups at 12-months does not necessarily mean that the educational intervention does not offer added benefit over usual care (as shown at 6-months), it may simply mean that the educational intervention needs to be repeated after 6-months because either the information and the behaviour change techniques are not retained in the longer term by some patients or more likely, some patients need these to be reinforced. The TREAT intervention is not simply about giving information it also gives patients a framework to make the necessary behavioural changes and strategies to maintain these over time. Therefore, a further ‘top-up’ of the intervention may be warranted six months after receiving the initial intervention, which may help to maintain the significantly improved levels of adherence. Indeed, a European-wide study of medication adherence identified that persistence with therapy is enhanced by interventions comprising education together with motivational and performance-based feedback and that such interventions may need to be repeated at intervals to support and reinforce behavioural change [34].

Patients’ knowledge scores at six months predicted TTR. This indicates that where patients’ knowledge regarding their illness and their treatment is sustained, patients are more likely to remain within target therapeutic range. The relationship between knowledge and adherence is unclear; however, it is possible that improving patient knowledge could reduce intentional and unintentional non-adherence [35]. Non-adherence is intentional when patients make a decision not to take their treatment as a result of their personal motivations or beliefs. Where these beliefs are inaccurate, or they perceive the barriers as too great, they are unlikely to adhere. Equally, improved knowledge of specific questions (e.g. ‘what should I do if I miss a dose of warfarin?’) could reduce unintentional non-adherence; which refers to an individual’s skills or ability to take their medications correctly (e.g. problems with remembering to take tablets). Evidence suggests that patients often report either or both types of non-adherence, with occasional overlap between the two concepts (e.g. where patients perceive medications as being unnecessary, they maybe more likely to forget to take it) [35,36]. Previous studies have also highlighted the link between knowledge and INR control [3,11].

There is a paucity of trialled theory-based interventions, despite the overwhelming evidence and guidelines to support their use [17,35,37]. For example, previous evidence has highlighted the link between patients’ beliefs about their medication and adherence but few interventions have addressed this potential link. By targeting those beliefs, and potentially improving adherence to medication, we may subsequently improve clinical outcomes.

Where patients view their medication as harmful, they are less likely to adhere [23,35,38]. This has been related to perceived ‘toxicity’ of medications, and patients’ views surrounding the impact of side effects in the short- and long- term [35]. Indeed, the present study found significant differences between the intervention and usual care group in their perception of medication harm in general and a significant interaction between general harm scores across time and between groups. It seems an obvious assumption that perceiving medications as harmful represents a barrier to adherence, and yet this is rarely considered in intervention design. Patients must undergo a personal risk evaluation when choosing to start a new medication, perhaps taking into consideration potential side effects (i.e. bleeding and bruising), perceived toxicity/potency of medication and risk reduction associated with warfarin [35]. This procedure is reliant on their ability to balance the risks associated with their treatment with those associated with their condition (i.e. stroke risks associated with AF vs. bleeding risks associated with warfarin).

Many AF patients may have preconceived ideas about how harmful warfarin is for example patients are more willing to take warfarin when they are blinded to the name of the treatment [39,40], highlighting the negative connotations associated with warfarin (e.g., ‘rat-poison). It is important that patients do not rely on inaccurate perceptions of harm, and that their risk evaluation draws upon reliable knowledge. The TREAT findings suggest that by reducing patients’ perception of harm, it may be possible to increase adherence levels. The inclusion of ‘expert’ patient narratives discussing their own experiences of bleeding and bruising in the DVD allowed the intervention group to assimilate this risk information into their own belief system.

There were also significant differences between the intervention and usual care groups’ perception of specific concern about their AF medication. Previous evidence suggests that those patients scoring higher on the specific concern subscale are less likely to adhere to medication [36,38,41]. This could provide some explanation as to why the intervention group spent more time in therapeutic range, although results do not suggest a causal link between specific concerns and TTR.

Using the sub-scales of the beliefs about medication questionnaire, it is possible to calculate a necessity-concerns differential score. This represents the difference between patients’ concerns about their AF medication and their perception of its necessity and there were significant differences across time and between groups. The intervention group scored higher, suggesting these patients perceived the necessity for warfarin as more important than their concerns about taking it. Baseline differential scores significantly correlated with TTR at six months, suggesting those patients with lower scores on specific concern, and higher scores on specific necessity (thus higher differential scores), also spent more time in the therapeutic range. This supports previous evidence with depressed patients taking selective serotonin reuptake inhibitors, whereby high necessity and low concern scores surrounding their anti-depressants were associated with greater self-reported adherence [42].

### Limitations

The majority of the TREAT participants were of white ethnicity, which does not reflect the multi-ethnic community of the West Midlands; but it does reflect the disease prevalence, as AF is predominantly seen in white populations [43]. However, the intervention is applicable to all ethnic groups and could be easily adapted into a range of languages, and to be culturally sensitive (e.g. including specific dietary requirements). The recruitment target was 78 patients in each group (allowing for a 20% attrition rate over time) however, 234 (36.2%) eligible patients declined to participate primarily due to the questionnaire burden; the decline rate was higher than we had anticipated resulting in only 97 patients being recruited. This trial may be limited somewhat by its small sample size however, other similar trials have also demonstrated significant differences in outcomes between groups with comparable samples [11], although it is possible that some relationships between factors studied remained undetected, due to a lack of statistical power. In addition, given the questionnaire burden, a large proportion of the sample failed to complete all questionnaires at every time point which resulted in the analyses of the psychological variables over time to elucidate factors which might explain the significant difference in TTR at 6 months between groups being exploratory, as they could only be undertaken in those who completed the questionnaires at every time point. Although the number of patients completing questionnaires at each time point fell over time, the proportion was similar in the intervention and usual care arms over the first six months. However, it was significantly lower in the intervention group at 12 months therefore, since the exploratory analyses only included those who had completed the 12 month set, these results may not be representative of the whole cohort.

Whilst novel OAC provides an alternative treatment, without the inconvenience of INR monitoring and lifestyle changes, there is currently some resistance to prescribing them due in part to the cost and the lack of ‘real-life’ efficacy and safety data, compared to warfarin. Therefore, warfarin is likely to remain as a treatment option for stroke prevention in AF. In addition, patient preferences for OAC treatment are also important in facilitating a shared decision-making process and many patients may still choose warfarin or prefer to remain on warfarin, or be unable or unwilling to take the new OACs [6]. Clinicians are often reluctant to prescribe warfarin due to patients’ lack of knowledge surrounding OAC and patients’ management of factors that might affect INR control [4]. The benefit of this intervention in terms of improvement in TTR may help to alleviate some of these fears, increase uptake and adherence, and translate into fewer adverse outcomes. Thus a one-off intensive intervention package provides a cost-effective alternative in improving INR control, and this intervention could be adapted for use with novel oral anticoagulant drugs.

## Conclusion

The TREAT intervention provides a simple one-off behavioural education session, which significantly improves adherence to warfarin as evidenced by greater TTR compared to usual care. Improving patient understanding surrounding AF, treatment necessity and stroke risk reduction, facilitates informed decisions about the management of their condition and treatment and can also make a significant difference to long-term adherence. The intervention’s ability to improve adherence highlights the importance of AF patients’ perceptions of the necessity of their treatment and clinical outcomes. Whilst novel oral anticoagulants are likely to become more widely available, removing the need for INR monitoring, warfarin will still remain as a widely used treatment option. Educating patients remains important regardless of the type of anticoagulation (VKA or novel OAC) to ensure patients are managing their treatment appropriately.

## Supporting Information

Figure S1 **Illness perceptions over time stratified by randomisation group among patients who completed all questionnaires at all time points.**
(TIF)

Figure S2 **Quality of life over time stratified by randomisation group among patients who completed all questionnaires at all time points.**
(TIF)

Figure S3 **Beliefs about medication over time stratified by randomisation group among patients who completed all questionnaires at all time points.**
(TIF)

Figure S4 **Anxiety and depression levels over time stratified by randomisation group among patients who completed all questionnaires at all time points.**
(TIF)

Checklist S1 **CONSORT Checklist.**
(DOC)

Table S1 **Change in scores between baseline and six months and baseline and 12 months for psychological measures.**
(DOCX)

Table S2 **Number of patients completing each questionnaire at each time point by randomisation group.**
(DOCX)

Table S3 **Psychological measures from baseline to 12 months for those completing questionnaires at all time points.**
(DOCX)

Protocol S1 **Trial protocol.**
(PDF)
