# Supplementary figures and images for: Educational Intervention Improves Anticoagulation Control in Atrial Fibrillation Patients: The TREAT Randomised Trial
Source: PLoS One. 2013 Sep 9;8(9):e74037. doi: 10.1371/journal.pone.0074037 (PMC3767671; doi:10.1371/journal.pone.0074037)

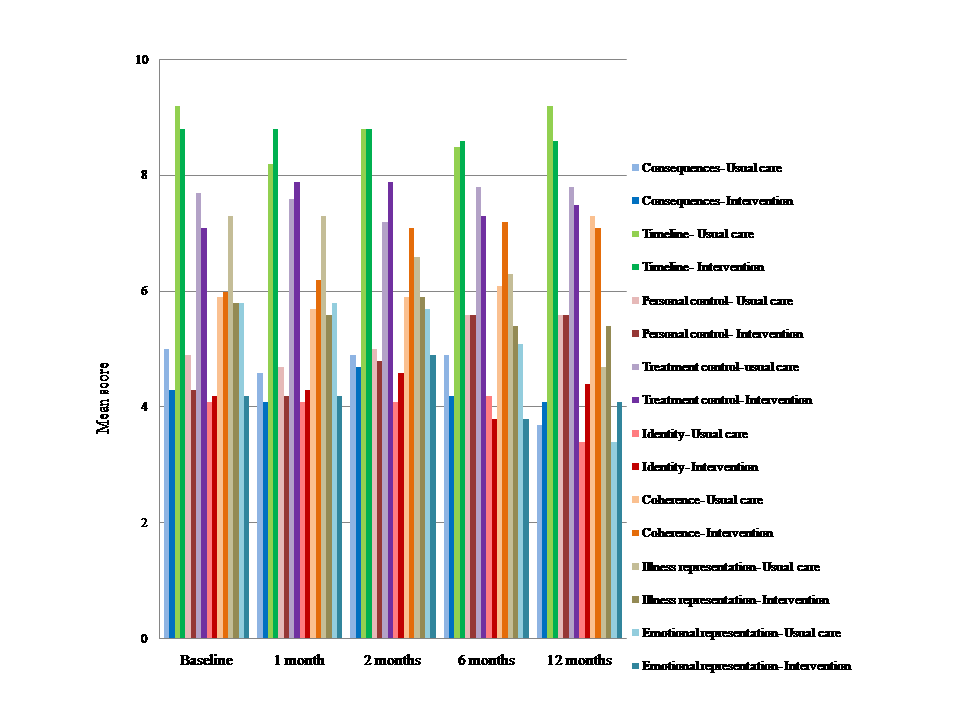

Supplement: Figure S1 — Illness perceptions over time stratified by randomisation group among patients who completed all questionnaires at all time points. (TIF) [file pone.0074037.s001.tif]

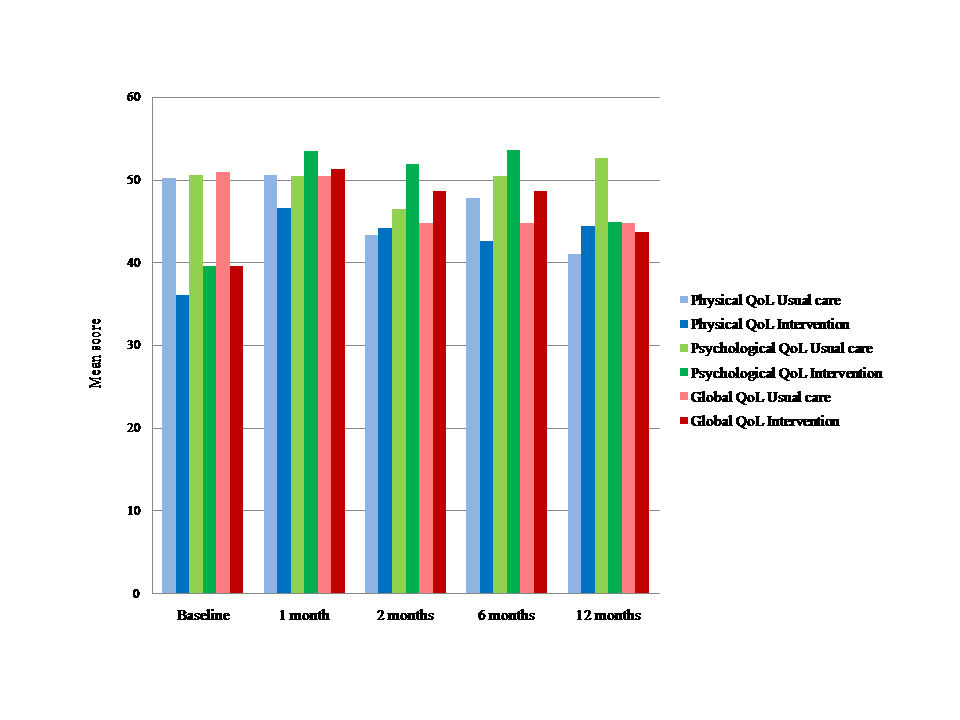

Supplement: Figure S2 — Quality of life over time stratified by randomisation group among patients who completed all questionnaires at all time points. (TIF) [file pone.0074037.s002.tif]

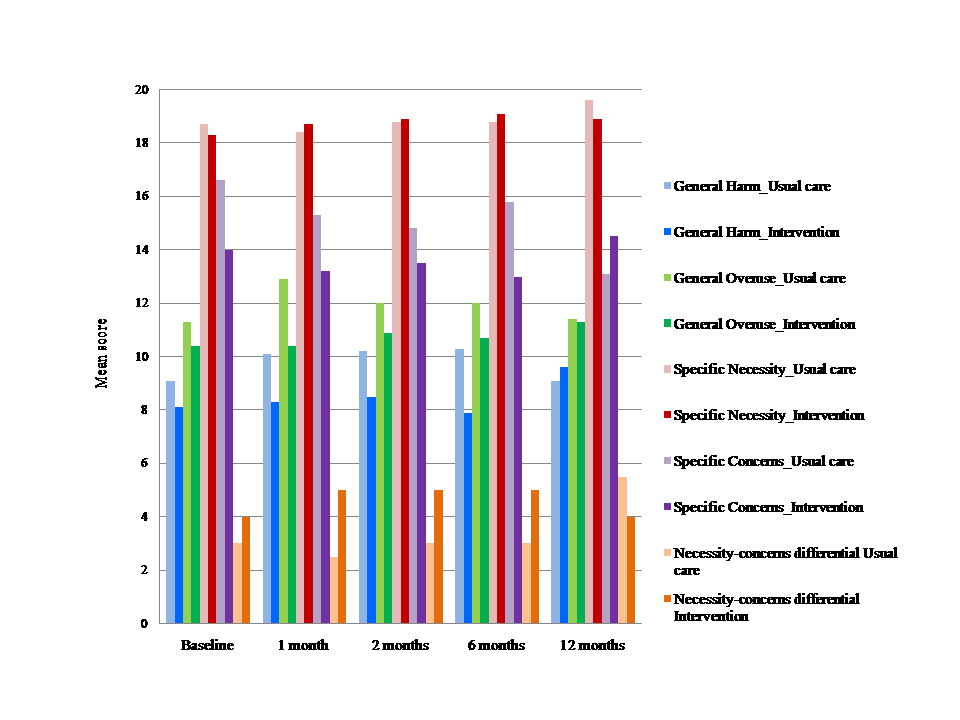

Supplement: Figure S3 — Beliefs about medication over time stratified by randomisation group among patients who completed all questionnaires at all time points. (TIF) [file pone.0074037.s003.tif]

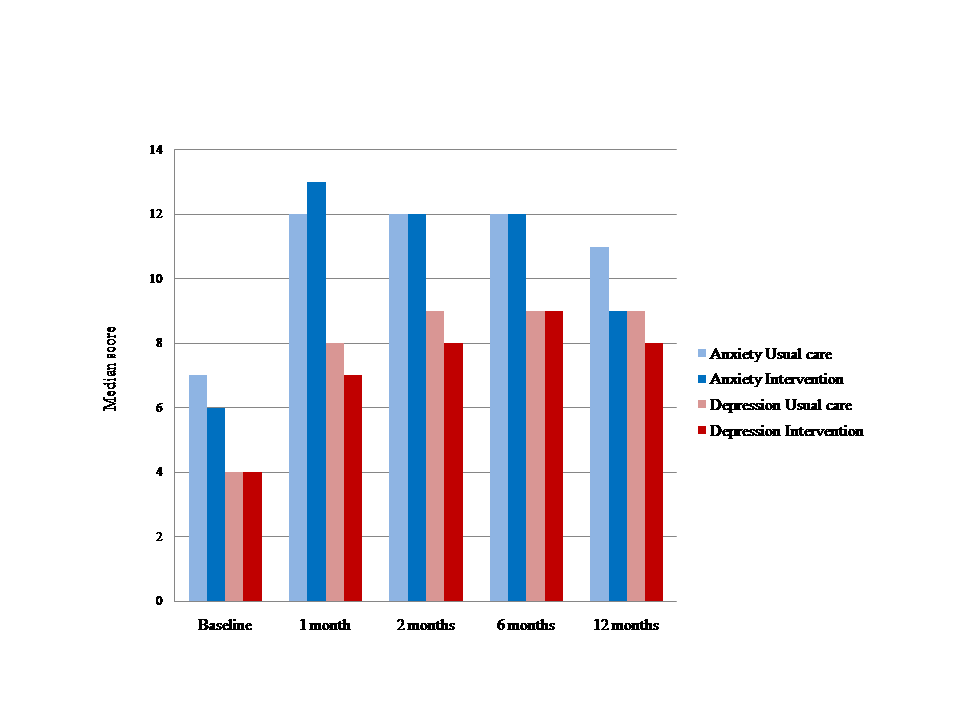

Supplement: Figure S4 — Anxiety and depression levels over time stratified by randomisation group among patients who completed all questionnaires at all time points. (TIF) [file pone.0074037.s004.tif]
