# Supplementary material for: Educational Intervention Improves Anticoagulation Control in Atrial Fibrillation Patients: The TREAT Randomised Trial
Source: PLoS One. 2013 Sep 9;8(9):e74037. doi: 10.1371/journal.pone.0074037 (PMC3767671; doi:10.1371/journal.pone.0074037)
Supplement: Table S1 — Change in scores between baseline and six months and baseline and 12 months for psychological measures. (DOCX) [file pone.0074037.s006.docx]

**Table S1:** Change in scores between baseline and six months and baseline and 12 months for psychological measures

| Median (IQR) change in score | **Baseline to 6 months** | | **Baseline to 12 months** | |
| --- | --- | --- | --- | --- |
|  | **Intervention** | **Usual care** | **Intervention** | **Usual care** |
| **Illness representations** | n=29 | n=31 | n=21 | n=31 |
| Consequences | 0 (-1.0 to 1.0) | 0 (-1.0 to 1.0) | 1.0 (-4.0 to 3.5) | 1.0 (-1.0 to 4.0) |
| Timeline | 0 (0 to 0.5) | 0 (0 to 1.0) | 0 (0 to 1.0) | 0 (-1.0 to 0) |
| Personal control | 0 (-2.0 to 1.0) | 0 (-2.0 to 2.0) | -2.0 (-4.0 to 1.0) | -1.0 (-4.0 to 2.0) |
| Treatment control | -1.0 (-2.5 to 0.5) | 0 (-2.0 to 2.0) | 0 (-2.0 to 1.5) | 0 (-3.0 to 1.0) |
| Identity | 0 (-0.5 to 2.0) | 0 (0 to 2.0) | 0 (-3.0 to 3.0) | 1.0 (-3.0 to 5.0) |
| Coherence | 0 (-0.5 to 2.0) | 0 (-1.0 to 2.0) | 0 (-2.5 to 4.5) | 0 (-2.0 to 6.0) |
| Illness concern | 0 (-1.5 to 2.0) | 0 (-1.0 to 1.0) | 2 (-3.5 to 5.0) | 2.0 (0 to 5.0) |
| Emotional representation | -1.0 (-3.0 to 0) | 0 (-2.0 to 2.0) | 0-1.0 (-3.5 to 1.0) | -3.0 (-4.0 to 0) |
| **Beliefs about medication** | n=29 | n=32 | n=22 | n=32 |
| General harm | 0 (-1.0 to 1.5) | 0 (-2.0 to 1.0) | 0.5 (-2.0 to 2.25) | 1.0 (-1.5 to 2.5) |
| General overuse | 0 (-2.0 to 1.0) | 0 (-2.0 to 1.0) | 0 (-2.50 to 2.50) | -1.0 (-3.5 to 2.0) |
| Specific necessity | -1.0 (-3.0 to 1.0) | 0.5 (-2.0 to 2.0) | -1.5 (-3.25 to 3.75) | -1.0 (-3.75 to 2.0) |
| Specific concern | 1.0 (-1.5 to 3.0) | 1.0 (-3.75 to 3.75) | -1.0 (-3.25 to 2.50) | 4.0 (-2.5 50 6.75) |
| Necessity-concerns differential | 0 (-5.0 to 1.0) | -1.0 (-5.0 to 3.50) | -1.0 (-4.25 to 4.0) | -4.0 (-10.0 to 3.0) |
| **HADS** | n=28 | n=28 | n=18 | n=30 |
| Anxiety | -7.5 (-11.0 to -3.0) | -4.5 (-9.2 to 0) | -4.0 (-5.0 to 2.25) | -2.0 (-7.0 to 1.5) |
| Depression | -4.0 (-7.0 to -2.0) | -4.5 (-6.0 to -2.0) | -3.50 (-5.25 to 0.50) | -4.0 (-7.0 to 0) |
| **Quality of life** | n=29 | n=30 | n=20 | n=30 |
| Physical QoL | 0 (-12.5 to 3.1) | -3.1 (-10.9 to 13.3) | -9.4 (-21.1 to 12.5) | 9.4 (-21.1 to 41.4) |
| Psychological QoL | -12.5 (-32.1 to 2.7) | -7.1 (-14.3 to 10.7) | 0 (-28.6 to 16.1) | 0 (-29.5 to 22.3) |
| Global QoL | -4.2 (-15.3 to 5.6) | 1.4 (-11.1 to 13.5) | -6.25 (-13.9 to 8.0) | 12.5 (-22.6 to 28.8) |

† Median (IQR) is reported where data is not normally distributed
