# Supplementary material for: Educational Intervention Improves Anticoagulation Control in Atrial Fibrillation Patients: The TREAT Randomised Trial
Source: PLoS One. 2013 Sep 9;8(9):e74037. doi: 10.1371/journal.pone.0074037 (PMC3767671; doi:10.1371/journal.pone.0074037)
Supplement: Table S2 — Number of patients completing each questionnaire at each time point by randomisation group. (DOCX) [file pone.0074037.s007.docx]

**Table S2:** Number of patients completing each questionnaire at each time point by randomisation group

|  | **Baseline** | | **1 month** | | **2 months** | | **6 months** | | **12 months** | |
| --- | --- | --- | --- | --- | --- | --- | --- | --- | --- | --- |
| N (% by randomisation) | Intervention | Usual care | Intervention | Usual care | Intervention | Usual care | Intervention | Usual care | Intervention | Usual care |
| Illness Perceptions Questionnaire | 46 (100) | 49 (96.1) | 32 (69.6) | 39 (76.5) | 26 (56.5) | 31 (60.8) | 29 (63.0) | 31 (60.8) | 21 (45.7) | 31 (60.7)* |
| Beliefs about Medication Questionnaire | 46 (100) | 50 (98.0) | 32 (69.6) | 40 (78.4) | 27 (58.7) | 30 (58.8) | 29 (63.0) | 32 (62.7) | 22 (47.8) | 33 (64.7)* |
| AF-Quality of Life | 46 (100) | 49 (96.1) | 32 (69.6) | 40 (78.4) | 27 (58.7) | 28 (54.9) | 27 (58.7) | 28 (54.9) | 21 (45.7) | 30 (58.8)* |
| Hospital Anxiety and Depression Scale | 44 (95.7) | 50 (98.0) | 32 (69.6) | 40 (78.4) | 25 (54.3) | 29 (56.9) | 28 (60.9) | 28 (54.9) | 18 (39.1) | 33 (64.7)* |
|  |  |  |  |  |  |  |  |  |  |  |
