# Supplementary material for: Educational Intervention Improves Anticoagulation Control in Atrial Fibrillation Patients: The TREAT Randomised Trial
Source: PLoS One. 2013 Sep 9;8(9):e74037. doi: 10.1371/journal.pone.0074037 (PMC3767671; doi:10.1371/journal.pone.0074037)
Supplement: Table S3 — Psychological measures from baseline to 12 months for those completing questionnaires at all time points. (DOCX) [file pone.0074037.s008.docx]

**Table S3:** Psychological measures from baseline to 12 months for those completing questionnaires at all time points

|  | **Baseline** | | **1 month** | | **2 months** | | **6 months** | | **12 months** | |
| --- | --- | --- | --- | --- | --- | --- | --- | --- | --- | --- |
|  | **Intervention** | **Usual care** | **Intervention** | **Usual care** | **Intervention** | **Usual care** | **Intervention** | **Usual care** | **Intervention** | **Usual care** |
| **IPQ** (n=27) | n=14 | n=13 |  |  |  |  |  |  |  |  |
| Consequences | 4.3 (2.3) | 5.0 (2.5) | 4.1 (2.6) | 4.6 (2.7) | 4.7 (2.8) | 4.9 (2.4) | 4.2 (2.8) | 4.9 (2.5) | 4.1 (3.3) | 3.7 (2.3) |
| Timeline | 8.8 (2.5) | 9.2 (1.7) | 8.8 (2.1) | 8.2 (2.7) | 8.8 (1.9) | 8.8 (1.9) | 8.6 (2.5) | 8.5 (2.6) | 8.6 (2.3) | 9.2 (1.3) |
| Personal control | 4.3 (3.1) | 4.9 (3.1) | 4.2 (2.9) | 4.7 (2.6) | 4.8 (2.8) | 5.0 (2.7) | 5.6 (2.9) | 5.6 (2.9) | 5.6 (2.7) | 5.6 (2.8) |
| Treatment control | 7.1 (2.3) | 7.7 (2.3) | 7.9 (1.9) | 7.6 (1.7) | 79 (1.6) | 7.2 (1.8) | 7.3 (1.9) | 7.8 (2.1) | 7.5 (2.0) | 7.8 (1.9) |
| Identity | 4.2 (2.7) | 4.1 (2.9) | 4.3 (3.0) | 4.1 (2.7) | 4.6 (2.8) | 4.1 (2.6) | 3.8 (2.8) | 4.2 (3.1) | 4.4 (3.2) | 3.4 (2.3) |
| Coherence | 6.0 (7.8) | 5.9 (2.9) | 6.2 (2.6) | 5.7 (3.1) | 7.1 (2.4) | 5.9 (2.9) | 7.2 (2.3) | 6.1 (2.8) | 7.1 (2.3) | 7.3 (2.4) |
| Emotional representation | 4.2 (2.7) | 5.8 (3.3) | 4.2 (2.9) | 5.8 (3.3) | 4.9 (3.0) | 5.7 (3.1) | 3.8 (2.9) | 5.1 (3.1) | 4.1 (3.1) | 3.4 (2.9) |
| Illness concern | 5.8 (3.1) | 7.3 (3.1) | 5.6 (3.1) | 7.3 (2.9) | 5.9 (2.9) | 6.6 (3.1) | 5.4 (3.1) | 6.3 (3.1) | 5.4 (3.3) | 4.7 (2.8) |
| **AF-QoL** (n=25) | n=12 | n=13 |  |  |  |  |  |  |  |  |
| Physical QoL | 36.1 (26.4) | 50.2 (27.3) | 46.6 (26.2) | 50.6 (29.9) | 44.1 (25.9) | 43.3 (25.2) | 42.6 (20.9) | 47.8 (26.9) | 44.4 (27.9) | 41.0 (24.2) |
| Psychological QoL | 39.5 (20.8) | 50.6 (26.3) | 53.5 (24.9) | 50.5 (28.8) | 51.9 (25.2) | 46.4 (29.2) | 53.6 (24.9) | 50.5 (28.8) | 44.9 (24.2) | 52.6 (24.2) |
| Global QoL | 39.6 (20.2) | 50.9 (22.6) | 51.3 (21.8) | 50.4 (24.8) | 48.6 (21.8) | 44.7 (23.3) | 48.6 (21.8) | 44.7 (23.3) | 43.7 (22.1) | 44.8 (19.9) |
| **BMQ** (n=29) | n=14 | n=15 |  |  |  |  |  |  |  |  |
| General Harm | 8.1 (2.1) | 9.1 (2.2) | 8.3 (2.1) | 10.1 (2.9) | 8.5 (1.7) | 10.2 (2.5) | 7.9 (2.0) | 10.3 (2.6) | 9.6 (2.6) | 9.1 (2.6) |
| General overuse | 10.4 (3.0) | 11.3 (2.9) | 10.4 (3.7) | 12.9 (2.9) | 10.9 (3.4) | 12.0 (2.9) | 10.7 (3.5) | 12.0 (2.6) | 11.3 (3.3) | 11.4 (2.8) |
| Specific necessity | 18.3 (3.7) | 18.7 (4.2) | 18.7 (3.5) | 18.4 (3.9) | 18.9 (3.6) | 18.8 (3.4) | 19.1 (3.2) | 18.8 (4.1) | 18.9 (3.8) | 19.6 (3.5) |
| Specific concern | 14.0 (3.7) | 16.6 (4.2) | 13.2 (3.4) | 15.3 (2.3) | 13.5 (3.5) | 14.8 (4.6) | 13.0 (3.9) | 15.8 (5.3) | 14.5 (4.5) | 13.1 (3.8) |
| Necessity-concerns differential† | 4.0 (1-8) | 3.0 (-1-5.5) | 5.0 (2-8) | 2.5 (0.25-6) | 5.0 (1-10) | 3.0 (0-7) | 5.0 (1.5-9.5) | 3.0 (-1-5.75) | 4.0 (1.2-8.7) | 5.5 (4-10.2) |
| **HADS** (n=23) | n=9 | n=14 |  |  |  |  |  |  |  |  |
| Anxiety Score† | 6 (3.7-9) | 7 (3-10.7) | 13 (11-13.·7) | 12 (10-14) | 12 (10-14) | 12 (9-14) | 12 (11-14) | 12 (10-13.7) | 9 (7-12) | 11 (9-12.7) |
| Depression score† | 4 (2-7) | 4 (2-8) | 7 (5-9) | 8 (7-9) | 8 (8-9) | 9 (8-10) | 9 (8-9) | 9 (8-10) | 8 (6-9·5) | 9 (7-10) |

Mean (SD) is reported where appropriate; † Median (IQR) is reported where data is not normally distributed

AF-QoL = Atrial fibrillation quality of life; BMQ = Beliefs about medication questionnaire; HADS = Hospital Anxiety and Depression; IPQ = Illness Perception Questionnaire
