# Supplementary material for: Educational Intervention Improves Anticoagulation Control in Atrial Fibrillation Patients: The TREAT Randomised Trial
Source: PLoS One. 2013 Sep 9;8(9):e74037. doi: 10.1371/journal.pone.0074037 (PMC3767671; doi:10.1371/journal.pone.0074037)
Supplement: Protocol S1 — Trial protocol. (PDF) [file pone.0074037.s009.pdf]

Smith et al. BMC Cardiovascular Disorders 2010, 10:21
http://www.biomedcentral.com/1471-2261/10/21

BMC Cardiovascular Disorders

STUDY PROTOCOL Open Access

TRial of an Educational intervention on patients' knowledge of Atrial fibrillation and anticoagulant therapy, INR control, and outcome of Treatment with warfarin (TREAT)

Danielle E Smith[1,2], Christian Borg Xuereb[1,2], Helen M Pattison[2], Gregory YH Lip[1,2] and Deirdre A Lane*[1,2]

Abstract

Background: Atrial fibrillation (AF) patients with a high risk of stroke are recommended anticoagulation with warfarin. However, the benefit of warfarin is dependent upon time spent within the target therapeutic range (TTR) of their international normalised ratio (INR) (2.0 to 3.0). AF patients possess limited knowledge of their disease and warfarin treatment and this can impact on INR control. Education can improve patients' understanding of warfarin therapy and factors which affect INR control.

Methods/Design: Randomised controlled trial of an intensive educational intervention will consist of group sessions (between 2-8 patients) containing standardised information about the risks and benefits associated with OAC therapy, lifestyle interactions and the importance of monitoring and control of their International Normalised Ratio (INR). Information will be presented within an 'expert-patient' focussed DVD, revised educational booklet and patient worksheets. 200 warfarin-naïve patients who are eligible for warfarin will be randomised to either the intervention or usual care groups. All patients must have ECG-documented AF and be eligible for warfarin (according to the NICE AF guidelines). Exclusion criteria include: aged < 18 years old, contraindication(s) to warfarin, history of warfarin USE, valvular heart disease, cognitive impairment, are unable to speak/read English and disease likely to cause death within 12 months. Primary endpoint is time spent in TTR. Secondary endpoints include measures of quality of life (AF-QoL-18), anxiety and depression (HADS), knowledge of AF and anticoagulation, beliefs about medication (BMQ) and illness representations (IPQ-R). Clinical outcomes, including bleeding, stroke and interruption to anticoagulation will be recorded. All outcome measures will be assessed at baseline and 1, 2, 6 and 12 months post-intervention.

Discussion: More data is needed on the clinical benefit of educational intervention with AF patients receiving warfarin.

Trial registration: ISRCTN93952605

Background

Atrial fibrillation (AF) is the most common arrhythmia in clinical practice, for people aged 40 years and older the lifetime risk of developing AF is approximately 25% [1]. AF is an independent risk factor for stroke, conferring a five-fold excess risk in AF patients compared to those in sinus rhythm [2] and accounts for almost 10-15% of all ischaemic strokes and approximately one in four strokes in those aged over 80 years [2]. Furthermore, when a stroke occurs in association with AF, patients have substantially greater mortality, morbidity, disability and longer hospital stays [3].

Despite the overwhelming evidence that thromboprophylaxis with warfarin substantially reduces the incidence of stroke and mortality compared to placebo [4] and is associated with a marked reduction in all strokes when compared to aspirin [4], it remains under-utilised [5]. Dose-adjusted warfarin does not confer a significant increased risk of bleeding [6], however, poor anticoagulation control, evidenced by a 10% rise in time out of INR range (2.0 to 3.0), was significantly associated with an

* Correspondence: deirdre.lane@swbh.nhs.uk
[1] University of Birmingham Centre for Cardiovascular Sciences, City Hospital, Dudley Road, Birmingham, B18 7QH, UK
Full list of author information is available at the end of the article

BioMed Central
© 2010 Smith et al; licensee BioMed Central Ltd. This is an Open Access article distributed under the terms of the Creative Commons Attribution License (http://creativecommons.org/licenses/by/2.0), which permits unrestricted use, distribution, and reproduction in any medium, provided the original work is properly cited.

increased risk of death (OR, 1.29 [95% CI, 1.23 to 1.36]), ischaemic stroke (OR, 1.10 [1.03 to 1.18]), and thromboembolic events (OR, 1.12 [1.06 to 1.19]) [7]. Recent analyses have demonstrated that the minimum threshold of time spent in the therapeutic INR range for warfarin to be efficacious is ≥ 58%, although a TTR >65% is associated with a 2.29% (95% CI 1.57-3.35; $p < 0.0001$) reduction in stroke risk [8]. Analyses from an observational UK cohort of AF patients [9] found that 51% of patients at high risk of stroke ($CHADS_2$ score ≥ 2) were outside the therapeutic range for 50% or more of the time for the duration of their warfarin treatment [9] and that TTR needed to be >71% for warfarin to be efficacious [9].

Patients' illness representations and their beliefs about their health are crucial determinants of whether anticoagulant treatment, particularly with warfarin, is successful [10-12]. Patients need to clearly understand the causes and consequences of their AF, as well as the risks and benefits of warfarin therapy, and incorporate this complex medical information into their own belief system. Studies suggest where patients have a greater knowledge of warfarin therapy, INR values are more often within target range ($p = 0.024$) [13]. However, patients often exhibit limited knowledge of their condition and their anticoagulant therapy [13-16]. It is important that interventions specifically target this group of patients to increase TTR and maximise clinical outcomes with warfarin.

Behavioural and educational interventions that targeted patients receiving oral anticoagulant (OAC) therapy, the majority for AF (other indications included deep vein thrombosis, prosthetic heart valves etc), found reduced incidence of major bleeding and mortality and increased TTR (e.g. Khan et al [17] found TTR 61.1% prior to intervention vs. 70.4% post intervention; mean difference 8.8; 95% CI: -0.2-17.8; $p = 0.054$), when compared to usual care [17,18]. Our earlier pilot study exclusively with AF patients [14] demonstrated a significant improvement in the awareness of target therapeutic INR ($p < 0.0001$) and factors which may affect INR levels ($p = 0.005$), with a trend towards improvement in the awareness of the benefits of anticoagulants and bleeding risks, six weeks after a brief educational intervention. Patients need key information about their condition and treatment to actively participate in their treatment management.

Methods/Design

TREAT is a '**TR**ial of an **E**ducational intervention on patients' knowledge of **A**trial fibrillation and anticoagulant therapy, INR control, and outcome of **T**reatment with warfarin'. It is a randomised controlled trial (RCT [ISRCTN93952605]) of an educational intervention versus usual care in a population of AF patients that have been newly prescribed anticoagulation treatment with warfarin. The project is funded by an investigator-initiated grant from Bayer Healthcare. The primary research question will examine the effects of an intensive educational intervention on patients' International Normalised Ratio (INR) control within the therapeutic range (INR 2.0 to 3.0) compared to patients receiving usual care. The secondary endpoints will determine the effects of the educational intervention on patients' knowledge of, and perceptions of AF and their beliefs about anticoagulant therapy. In addition, the relationship between INR control and the incidence of major and minor bleeding, stroke and thromboembolic events compared to patients receiving usual care will be explored.

To explore these endpoints TREAT will determine whether there are differences between the intervention group and the usual care group at baseline, 1, 2, 6 and 12 months post-intervention. Subgroup analysis of patient groups (using age, gender and socio-demographic data) will determine whether data outcomes relate to other factors such as the age or gender of the participants. Finally, a health-care utilisation assessment will be undertaken to determine the costs of the intensive educational intervention compared to usual care. This will determine whether the intervention would be cost effective if used on a larger scale as part of standard 'usual care'.

Patients

AF patients newly referred for warfarin therapy, with ECG-documented AF, will be eligible for inclusion. Patients will be excluded from participation if they are aged < 18 years old, have any contraindication to warfarin, have previously received warfarin, have valvular heart disease, are cognitively impaired, are unable to speak or read English and have any disease likely to cause their death within 12 months. Patients will be recruited from the outpatient AF clinics at City Hospital, Sandwell District General Hospital (part of SWBH NHS Trust) and Good Hope Hospital which collectively receive approximately 600 referrals for AF patients requiring anticoagulant prophylaxis per annum (see figure 1. for CONSORT recruitment flow diagram). Sandwell and West Birmingham NHS Trust serves a multi-ethnic inner-city population of approximately 600,000 people, while Good Hope Hospital serves North Birmingham, Sutton Coldfield, and a large part of South-East Staffordshire, with a catchment of 450,000.

Demographic and clinical information will be recorded at baseline from the patient's hospital records. Individualised annual risk of stroke will be determined based on risk stratification proposed by the NICE guidelines (low/moderate/high risk) [18] and the $CHADS_2$ scheme [19]. Patients who are eligible for OAC therapy will receive a standard explanation of the need for OAC therapy, and the risks/benefits of such treatment. Those who are

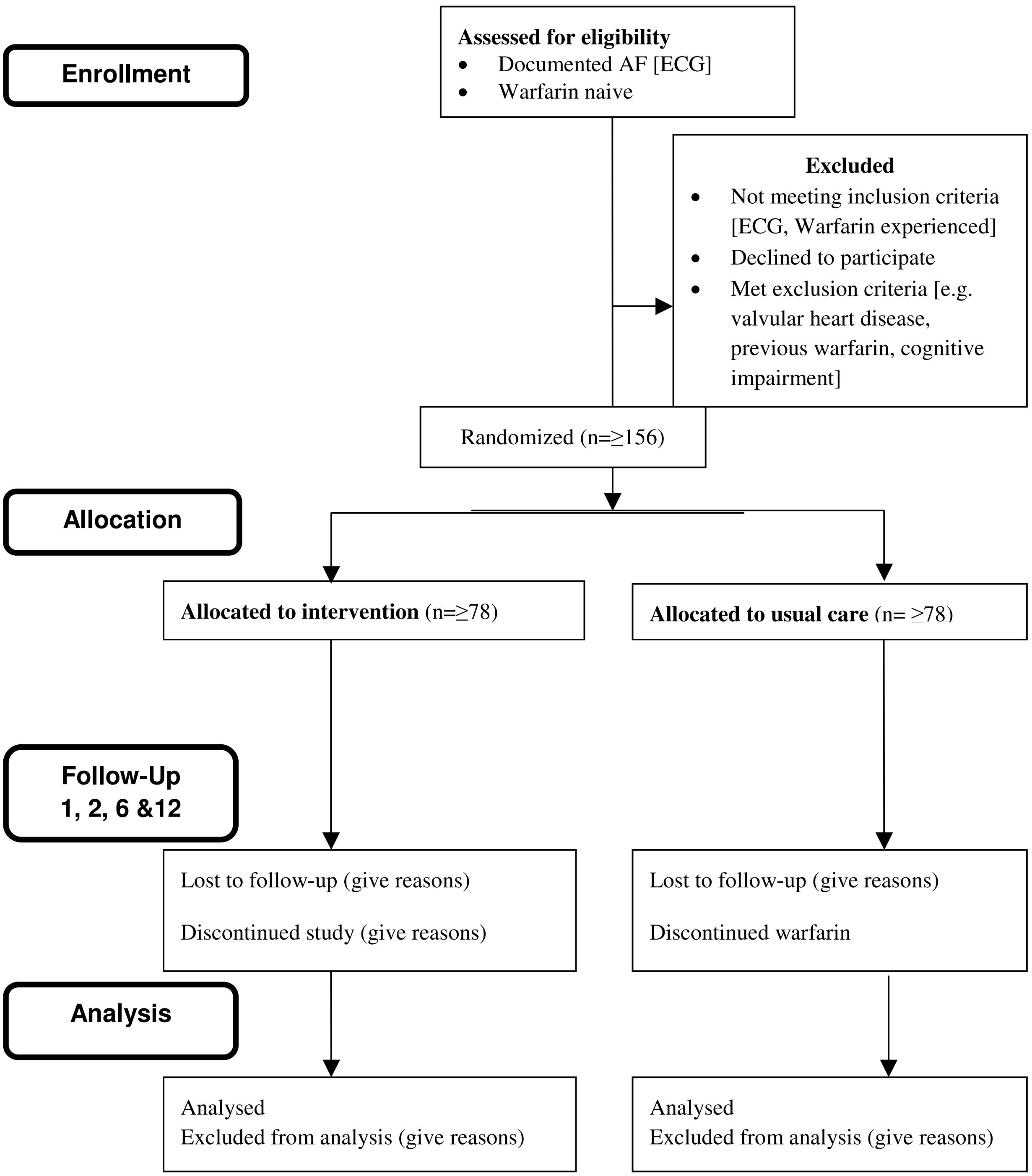

Figure 1 TREAT recruitment flow diagram

accepting of anticoagulant therapy will be approached to participate in this intervention study. Patients accepting of warfarin therapy and who consent to participate will be randomised to receive 'usual care' or the intensive intervention.

Ethics approval for the TREAT study has been granted by the Black Country Research Ethics Committee. All participants will provide written informed consent.

Randomisation procedure

At the stage of randomisation the primary researcher will check to ensure the patient meets the eligibility criteria and does not meet any of the exclusion criteria. If the patient is willing to take part consent, will be obtained prior to randomisation. A computer generated list will randomise patients in blocks of four and on an individual basis with stratification by (a) age [< 70 and 70+]/sex and (b) specialist AF clinic versus 'general' cardiology clinic. There is one specialist AF clinic vs. two general cardiology clinics involved in the trial. The specialist clinic is a large clinic receiving AF referrals from the entire SWBH Trust, therefore providing patient numbers equal to those provided by the general cardiology clinics. Patients will receive either 'usual care' (minimum of n = 78) or the intensive educational intervention (minimum of n = 78), in addition to 'usual care'. The randomisation schedule was designed by an independent trials unit and the random allocation will be obtained by the researcher telephoning an associate researcher (who is not involved in the data collection or data entry). The primary researcher will be blinded to patient identification to ensure allocation concealment. A third researcher will match patient identification numbers (generated by the primary researcher) with randomisation codes (generated by the associate researcher). Once the random allocation has been obtained baseline data will be collected and an intervention session will be arranged. Patients who refuse to be randomised will be offered their respective hospitals 'usual care' package.

Usual care

Patients randomised to usual care will be informed about their condition and the need for anticoagulant therapy only. All patients will also receive the standard information booklet which contains basic lifestyle recommendations pertaining to OAC therapy. When patients are first prescribed OAC, dosing officers and anticoagulation nurses use an educational checklist to ensure they have discussed all of the key information with the patients. This information includes the purpose of their treatment, future INR monitoring and factors which affect INR. This yellow patient information booklet contains some basic information pertaining to OAC therapy and their INR record book.

Intensive education

Those in the intensive educational intervention arm will attend one group session (between 2-8 patients) for one hour, where they will be shown a DVD of information about the need for warfarin, the risks and benefits associated with OAC therapy, potential interactions with food, drugs, and alcohol, and the importance of monitoring, and control of their INR. The information on the DVD will be communicated in a variety of ways [i.e. by expert patients, a cardiology consultant and examples of food/alcohol dietary components with educational information as a voiceover script].

The intervention session will be interactive, where the patients will be encouraged to ask questions following each 10 minute DVD section. Patients will also be asked to complete a worksheet-based exercise which will serve to reinforce the information presented. The chapters will focus on AF and treatment recommendations, INR and lifestyle recommendations and patient concerns about OAC [including frequently asked questions answered by the consultant cardiologist]. The content and format of the information presented was finalised after consultation with a focus group of AF patients currently taking OAC, and was based on the NICE guidelines for patients [18] to ensure that it is suitable for a wide-range of patients. In addition to the interactive group sessions, patients will also be given the revised educational booklet, which was originally developed for and employed in our pilot study [14]. The complete intervention pack contains a treatment diary whereby patients can self-monitor the factors which affect the stability of their INR, an alert card and their session worksheet. The content of the educational booklet will be covered in the group session and will serve to reinforce the information and enable the patient to refer to it in the future.

Study Outcomes

The *primary* endpoint is the proportion of time spent in the therapeutic INR range, 2.0 to 3.0. All patients (intervention and usual care groups) will attend the anticoagulant outpatient clinic to have their INR checked using a capillary blood sample. The frequency of the INR visits will be at the discretion of the OAC clinic. The OAC clinic staff will be blinded to the intervention arm the patient is randomised to, to enable as 'naturalistic' as possible follow-up and monitoring). Every INR result from baseline to the end of the study (12-months) will be recorded. The proportion of time each patient spends in the therapeutic INR range (2.0 to 3.0) will be calculated by the method of linear interpolation [7] using data from months 1 to 12 (to allow attainment of the correct dose of warfarin during the first four weeks).

Secondary endpoints include (1) patients' knowledge [an increase/decrease/or no change in knowledge will be

Table 1: Outcome measures for the TREAT study

Primary outcome measure	Secondary outcome measures
TTR of INR (analysed using methods defined by Connolly et al [8])	Patients' knowledge [14]
	Beliefs about medication (BMQ, [20])
	Illness representations (IPQ-B [22])
	Cost effectiveness [23]
	Hospital anxiety and depression [21]
	Minor and major bleeding incidence<br>Stroke incidence
	Thromboembolic events

measured by a change in score +/-/no change], (2) beliefs about medication [20] (3) anxiety and depression [21] and (4) illness representations ([22] see table 1). Ancillary analyses will explore the relationship between INR control and incidence of minor and major bleeding, stroke, thromboembolic events (given that the trial is not powered to detect these differences) and frequency of INR checks. The number of strokes, bleeding and thromboembolic events will be determined from the computerised clinical information system at the hospital. Patients in both arms of the trial will receive the follow-up questionnaires by post 1, 2, 6 and 12 months after commencing OAC therapy. If returned questionnaires are not fully completed, an independent researcher will contact the patient by telephone to facilitate 100% completion of the questionnaires.

Sample size

Power for the primary endpoint was calculated based on data from a secondary analysis of the time within therapeutic range (TTR) from the ACTIVE-W cohort by Connolly et al [8]. The power calculation assumes that usual care patients would have mean TTR of 58% with a standard deviation [SD] of 7.5. We hypothesise a 6% improvement in mean TTR in the intervention group (i.e. to 64%) with a similar SD (i.e. 7.5). In order for this improvement to be statistically significant with a 1-beta power of 0.99 and alpha = 0.01 we need a sample size of 154 subjects in two equal groups of 77 each. However, to ensure confidence and to allow for attrition, we intend to recruit in excess of this figure.

For the secondary endpoint of improvement in knowledge following the intervention, sample size was calculated based a study by Khan et al [17]. A sample size of 100 patients (allowing for a 20% attrition rate in completion of the questionnaires) will have at least 80% power to detect an 18.5% increase in knowledge about the condition and factors affecting INR control between baseline and follow-up.

Statistical analyses

All data will be analysed by intention to treat. All analyses will be stratified by centre. Comparisons with the primary and secondary outcome measures will be made at all time points to determine short and long term effects of the intervention. For continuous variables (e.g., changes in illness perception questionnaire or changes in time spent within INR therapeutic range), the weighted mean difference would be used. As the sample is randomised we would not expect baseline differences between groups.

Cost analysis

In addition to the cost of the intervention delivery (production, staffing and resource costs) and anticoagulation management, all patients will complete a questionnaire (previously employed by Jowett et al [23]) to evaluate their costs for two separate visits to the OAC clinic. Patient costs incurred when travelling to the clinic, including travel costs, other out of pocket expenses, loss of leisure time and net wage deduction associated with patients' clinic visits during working time will be collected. Additional societal costs also include the value of a companion's time.

Discussion

The TREAT trial seeks to recruit warfarin-naïve patients from cardiology clinics who are recommended for and accepting of OAC with warfarin. Providing an intensive educational intervention will determine whether increasing the information available to patients yields both clinical benefit and cost effectiveness. This is a pragmatic trial, thus limitations of the design are necessary for the initial trial of the intervention. For example, patients are not blinded to which arm of the trial they receive and results will not be generalisable to patients with cognitive impairment or limited English ability. However, if the trial is successful the intervention will be available for carers of patients with cognitive deficits and could potentially be translated and piloted in a range of other languages. Whilst the TREAT trial is powered for a surrogate outcome marker, TTR, clinical outcomes will be observed. TTR is an important outcome as INR control substantially reduces the risk of adverse events. Thus the TREAT study outcomes have implications for the management of all patients receiving OAC, as the intervention aims to improve knowledge on the key lifestyle factors which affect INR control and medication adherence. If the results are positive the educational intervention has potential for online dissemination and the materials are such that they can be delivered by a range of health-care

professionals, with suitable training. The final results of this study will not be available before late 2011.

Competing interests
The TREAT study is funded by an Investigator-Initiated Educational Grant from Bayer Healthcare.

Authors' contributions
DES, GYHL, and DAL conceived the idea for the trial, DES and DAL drafted the manuscript. HP, CBX and GYHL read and revised the manuscript. All authors contributed to the development of the intervention.

Author Details
[1]University of Birmingham Centre for Cardiovascular Sciences, City Hospital, Dudley Road, Birmingham, B18 7QH, UK and [2]School of Health and Life Sciences, Aston University, Aston Triangle, Birmingham, B4 7ET, UK

Received: 14 December 2009 Accepted: 20 May 2010
Published: 20 May 2010

References
1. Lloyd-Jones DM, Wang TJ, Leip EP: **Lifetime risk for developing atrial fibrillation: The Framingham Heart Study.** *Circulation* 2004, **110:**1042-1046.
2. Lip GYH, Edwards SJ: **Stroke prevention with aspirin, warfarin and ximelagatran in patients with non-valvular atrial fibrillation: A systematic review and meta-analysis.** *Thrombo Res* 2006, **110:**354-358.
3. Steger C, Pratter A, Martinek-Bregel M: **Stroke patients with atrial fibrillation have a worse prognosis than patients without: data from the Austrian Stroke Registry.** *Eur Heart J* 2004, **25:**1734-1740.
4. Hart RG, Pearce LA, Aguillar ML: **Meta-analysis: antithrombotic therapy to prevent stroke in patients who have nonvalvular atrial fibrillation.** *Ann Intern Med* 2007, **146:**857-867.
5. Nieuwlaat R, Capucci A, Lip GYH: **Antithrombotic treatment in real-life atrial fibrillation patients: a report from the Euro Heart Survey on Atrial Fibrillation.** *Eur Heart J* 2006, **27:**3018-3026.
6. van Walraven C, Hart RG, Singer DE: **Oral anticoagulants vs. aspirin in nonvalvular atrial fibrillation: an individual patient meta-analysis.** *JAMA* 2002, **288:**2441-2448.
7. Jones M, McEwan P, Morgan CL: **Evaluation of the pattern of treatment, level of anticoagulation control, and outcome of treatment with warfarin in patients with non-valvar atrial fibrillation: a record linkage study in a large British population.** *Heart* 2005, **91:**472-477.
8. Connolly SJ, Pogue J, Eikelboom J: **Benefit of oral anticoagulation over antiplatlet therapy in atrial fibrillation depends upon the quality of the international normalised ratio achieved by centres and countries as measured by time in therapeutic range.** *Cirulation* 2008, **118:**2029-2037.
9. Morgan CLI, McEwan P, Tukiendorf A: **Warfarin tratment in patients with atrial fibrillation: observing outcomes associated with varying levels of INR control.** *Thrombo Res* 2009, **124:**37-41.
10. Howitt A, Armstrong D: **Implementing evidence based medicine in general practice: audit and quality of antithrombotic treatment for atrial fibrillation.** *BMJ* 1999, **318:**1324-1327.
11. Protheroe J, Fahey T, Montgomery A: **The impact of patients' preferences on the treatment of atrial fibrillation: an observational study of patients bases decision analysis.** *BMJ* 2000, **320:**1380-1384.
12. Bungard TJ, Ghali WA, Teo KK: **Why do patients with atrial fibrillation not recieve warfarin.** *Arch of Intern Med* 2000, **160:**46-41.
13. Tang E, Lai C, Lee K: **Relationships between patients' warfarin knowledge and anticoagulation control.** *Ann Pharm* 2003, **37:**34-39.
14. Lane DA, Ponsford J, Shelley A: **Patient knowledge and perceptions of atrial fibrillation and anticoagulant therapy: Effects of an educational intervention programme The West Birmingham Atrial Fibrillation Project.** *Int J Cardiol* 2006, **110:**354-358.
15. Coelho-Dantas G, Thompson BV, Manson JA: **Patients' perspectives on taking warfarin: qualitative study in family practice.** *BMC Fam Pract* 2004, **5:**15.
16. Nadar S, Begum N, Kaur B: **Patients' understanding of anticoagulation therapy in multiethinic population.** *J Royal Soc Med* 2003, **96:**175-179.
17. Khan TI, Kamali F, Kesteven P: **The value of education and self-monitoring in the management of warfarin therapy in older patients with unstable control of anticoagulation.** *Br J Haematol* 2004, **126:**557-564.
18. NICE: *Atrial fibrillation: national clinical guideline for management in primary and secondary care* Royal College of Physicians, London; 2006.
19. Fuller R, Dudley N, Blacktop J: **Avoidance hierarchies and preferences for anticoagulation--semi-qualitative analysis of older patients' views about stroke prevention and the use of warfarin.** *Age Ageing* 2004, **33:**608-611.
20. Horne R, Weinman J, Hankins M: **The Beliefs about Medicines Questionnaire: The development and evaluation of a new method for assessing the cognitive representation of medication.** *Psychol Health* 1999, **14:**1-24.
21. Zigmond AS, Snaith RP: **The hospital anxiety and depression scale.** *Acta Psychiatrica Scandinavica* 1983, **67:**361-370.
22. Weinman J, Petrie KJ, Moss-Morris R: **The illness perception questionairre: a new method for assessing the cognitive representation of illness.** *Psychol Health* 1996, **11:**431-446.
23. Jowett S, Bryan S, Mahe I: **A multinational investigation of time and travelling costs in attending anticoagulation clinics.** *Value in Health* 2008, **11(2):**207-212.

Pre-publication history
The pre-publication history for this paper can be accessed here:
http://www.biomedcentral.com/1471-2261/10/21/prepub

doi: 10.1186/1471-2261-10-21
Cite this article as: Smith *et al.*, TRial of an Educational intervention on patients' knowledge of Atrial fibrillation and anticoagulant therapy, INR control, and outcome of Treatment with warfarin (TREAT) *BMC Cardiovascular Disorders* 2010, **10**:21

Submit your next manuscript to BioMed Central and take full advantage of:

- Convenient online submission
- Thorough peer review
- No space constraints or color figure charges
- Immediate publication on acceptance
- Inclusion in PubMed, CAS, Scopus and Google Scholar
- Research which is freely available for redistribution

Submit your manuscript at
www.biomedcentral.com/submit

BioMed Central
